# A numerical renormalisation group method for the analysis of critical spreading activity in spiking neural networks

**DOI:** 10.1186/1471-2202-14-S1-P297

**Published:** 2013-07-08

**Authors:** Thomas Greg Corcoran, Andrew Phillipedes, Thomas Nowotny

**Affiliations:** 1 Centre for Computational Neuroscience and Robotics, University of Sussex, Falmer, BN1 9RH, UK

## 

In recent years criticality has been used as a mechanism for understanding the presence of long-range correlations and intermittence in macroscopic neural activity.

Little work has been done to demonstrate the usefulness of this paradigm for understanding the statistics and dynamics of neural networks.

The explanation for self-similarity and universality is grounded in the renormalisation group (RG) theory of critical phenomena [1]. Until recently [2], this theory has not been leveraged for the inspection and understanding of neural network models or in neural data analysis.

Here, we present a range of statistics derived from a dynamic renormalisation group theory [3], shown to elucidate the nature of self-organised criticality in sandpile models, which we have operationalised for the numeric data analysis of spike trains.

The relationship between universality via self-similarity, the power-law exponents and a set of renormalised parameters is explored in the case of two automata models to demonstrate the application of the RG. In particular, we focus on the temporal profile of avalanches, the branching ratio and the avalanche size distribution and how these can be related to the renormalised parameters we study in these systems. Next, the same statistics are used to investigate the universality of a simplified integrate-and-fire model.

Finally we use these methods to assess the critical state on a number of generic neural network models which are used to study the balanced state of neocortex.
